# Marine Algae-Derived Bioactive Compounds: A New Wave of Nanodrugs?

**DOI:** 10.3390/md19090484

**Published:** 2021-08-26

**Authors:** Farid Menaa, Udari Wijesinghe, Gobika Thiripuranathar, Norah A. Althobaiti, Aishah E. Albalawi, Barkat Ali Khan, Bouzid Menaa

**Affiliations:** 1 Department of Internal Medicine and Nanomedicine, Fluorotronics-CIC, San Diego, CA 92037, USA; bmenaa@cic-fluorotronics.com; 2 Institute of Chemistry Ceylon, College of Chemical Sciences, Rajagiriya 10107, Sri Lanka; udariemalka@ichemc.edu.lk (U.W.); tgobika@ichemc.edu.lk (G.T.); 3 Biology Department, College of Science and Humanities, Shaqra University, Al Quwaiiyah 19257, Saudi Arabia; nalthobaiti@su.edu.sa; 4 Biology Department, Faculty of Science, University of Tabuk, Tabuk 71491, Saudi Arabia; ae.albalawi@ut.edu.sa; 5 Department of Pharmacy, Gomal University, Dera Ismail Khan 29050, Pakistan; barkat.khan@gu.edu.pk

**Keywords:** marine algae, nanotheranostics, bioactive compounds, innovation, alternative and complementary medicine, diabetes, neurodegenerative diseases, marine drugs

## Abstract

Marine algae are rich in bioactive nutraceuticals (e.g., carbohydrates, proteins, minerals, fatty acids, antioxidants, and pigments). Biotic (e.g., plants, microorganisms) and abiotic factors (e.g., temperature, pH, salinity, light intensity) contribute to the production of primary and secondary metabolites by algae. Easy, profitable, and sustainable recovery methods include novel solid-liquid and liquid-liquid extraction techniques (e.g., supercritical, high pressure, microwave, ultrasound, enzymatic). The spectacular findings of algal-mediated synthesis of nanotheranostics has attracted further interest because of the availability of microalgae-based natural bioactive therapeutic compounds and the cost-effective commercialization of stable microalgal drugs. Algal extracts can serve as stabilizing/capping and reducing agents for the synthesis of thermodynamically stable nanoparticles (NPs). Different types of nanotherapeutics have been synthesized using physical, chemical, and biological methods. Marine algae are a fascinating source of lead theranostics compounds, and the development of nanotheranostics has been linked to enhanced drug efficacy and safety. Indeed, algae are remarkable nanobiofactories, and their pragmatic properties reside in their (i) ease of handling; (ii) capacity to absorb/accumulate inorganic metallic ions; (iii) cost-effectiveness; and (iv) capacity of eco-friendly, rapid, and healthier synthesis of NPs. Preclinical and clinical trials shall enable to really define effective algal-based nanotherapies. This review aims to provide an overview of the main algal compounds that are nutraceuticals and that can be extracted and purified for nanotheranostic purposes.

## 1. Introduction

Over the last two decades, the synergy between engineering and medical science has opened novel frontiers in the field of nanotheranostics. Nanoformulation is one of the fastest developing platforms to overcome limitations in the use of peptide-based drugs [1,2]. The paramount advantages of NPs in the development of nanotheranostics have been linked to enhanced drug effectiveness in several fascinating ways, by increasing the (i) biocompatibility (safety); (ii) systemic bioavailability (half-life); (iii) solubility (drug delivery); (iv) biodistribution (sustained and controlled drug release in target tissues and cells); (v) biostability; (vi) versatility and ability to overcome sequential biological barriers (e.g., pump-mediated multidrug (MDR) resistance, sequestration by the mononuclear phagocytic system); (vii) large-scale production; (viii) simple usage (e.g., possibility of nanodrug administration by any possible route (e.g., per os, transdermal, topical, intravenous); (ix) functional efficiency (surface functionalization), while reducing the side effects (e.g., sufficiency of minimal applied nanodrug concentration) [1,2,3,4].

Different types of nanotherapeutics have been synthesized using physical, chemical, and biological methods [5,6,7,8,9,10,11,12]. Theranostic NPs are categorized into: (i) hybrid NPs (composed of different nanomaterials, such as metals, biopolymers, and lipids); (ii) multifunctional NPs (functionalized with targeting moieties and/or drugs); and (iii) multifunctional hybrid NPs (incorporating characteristics of (i) and (ii)) [7,10,12,13,14]. The most promising nanotheranostics are modifiable/functionalizable nanosystems that generally combine more than one composite with a core-shell structure [15]. Often, nanosystems are functionalized with biocompatible polymeric layers and/or targeting moieties, including contrast agents, which succeed in forming the interaction between the imaging and the therapeutic parts of NPs [10,13,15,16]. Moreover, surface functionalization of NPs allows active targeting of cells and/or a combination of light-based modalities [4,10,13,16]. Therefore, the fabrication of nanoplatforms with in vivo stability for efficient delivery of drugs or diagnostic markers to biological targets is of foremost importance, as they will be exposed to drastic conditions within the microenvironment (e.g., tumor) [14,15].

Although different NPs have been formulated as nanotheranostics, their safety concerns in humans have not been completely studied yet [4,15]. When designing nanotheranostics, the identification of major factors is crucial for clinical applications (e.g., imaging/contrast modalities, chemotherapy) [3,10,15,16,17]; this includes a deeper understanding of the mechanisms by which a given theranostic, ideally cost-effective, is (i) administered (to avoid premature release from the delivery system); (ii) cleared from the body; (iii) able to interact with the immune system; and (iv) defined as safe (e.g., by optimizing the dose levels, drug encapsulation, ligand conjugation efficiency and efficacy, drug administration frequencies, as well as by ensuring the reproducibility of the theranostics’ effects in vitro, ex vivo, and in vivo (e.g., clinical trials)) [4,14,18].

It is worth mentioning that nanoformulations vary according to chemical, biochemical, and physicochemical properties of nanomaterials (e.g., particle size and surface area) [3,4,10,14,17,19]. The enhanced biological and catalytic activity, mechanical property, melting point, optical absorption, and thermal and electrical conductivity of NPs have attracted much attention for their use as nanomedicines (e.g., in the treatment, diagnosis, monitoring, and control of biological systems) [1,10,18]. Among the different types of NPs, magnetic NPs (e.g., γ-iron(III) oxide (γ-Fe_3_O_4_)), noble metal NPs (MNPs) (e.g., silver (Ag), gold (Au), copper (Cu), palladium (Pd), and platinum (Pt)), as well as semiconductor NPs (e.g., cadmium sulfide (CdS), zinc oxide (ZnO), titanium oxide (TiO_2_), zinc sulfite (O_3_SZn), and silica/silicon dioxide (SiO_2_)) are widely used as nanotheranostics in the field of drug delivery and diagnostics [3,10,20,21,22]. Physicochemical synthesis of NPs is often cumbersome and costly with the release of harmful by-products, posing a high risk to living systems [1,10,17]. Therefore, the fountainhead of nanobiotechnology is focused on the fabrication of structurally well-defined, reproducibly synthesizable NPs from biodegradable and/or biocompatible materials such as lipids, polysaccharides, proteins, or peptides [7,18,19,21].

Recently, biological synthesis of NPs using bacteria, fungi, viruses, plants, and algae has emerged as a promising field [7,19,21,23] due to (i) the use of green energy for NPs assembly, which subsequently overcomes the environmental toxicity; (ii) the large-scale synthesis; (iii) different biocompounds present in bio-organisms that help to obtain safe NPs of different morphology/shape, size distribution, composition, and stability; (iv) cost-effectiveness; and (v) versatile usage in a wide range of activities encompassing the cosmetics, theranostics, food, and textile fields.

Phyconanotechnology is becoming an exciting and upcoming area with greater scope in the synthesis of algae-based NPs [7,10,19,23]. Algae are remarkable aquatic, photosynthetic nanobiofactories, characterized by being (i) a major under-exploited reservoir of cost-effective bioactive compounds and (ii) an excellent choice to explore for applications in the renewable energy, food, pharmaceutical, nutraceutical, and cosmetic industries; with (iii) a high growth rate in sea water or controlled conditions, (iv) an ease of handling, and (v) a capacity to absorb/accumulate inorganic metallic ions; while being able to synthesize NPs in an eco-friendly, rapid, and healthier way [7,21,23,24]. Besides these overall challenges and properties, it is worth noting that not only living, but also dead algae can be used for the synthesis of nanotheranostics [7,13,17].

Algae are known to be the largest primitive photoautotrophic and polyphyletic group of eukaryotes, which perform more than 50% of photosynthesis on this planet [24]. They are classified and primarily based on their morphological features, either as microalgae (i.e., unicellular, such as diatoms, or multicellular) or as macroalgae (sometimes referred to as seaweeds). Marine algae are classified into three major distinct classes based on the presence of specific pigments. Therefore, algae can be brown (Phaeophyta/Phaeophyceae, such as *Sargassum polycystum*, *Padina pavonica*, and *Cystophora moniliformis*); blue-green (Cyanophyta/Cyanophyceae, such as *Spirulina platensis*, *Chlorococcum humicola*, and *Chlorella vulgaris*); green (Chlorophyta/Chlorophyceae, such as *Chlamydomonas reinhardtii*, *Ulva fasciata*, and *Gracilaria edulis*); or red (Rhodophyta/Rhodophyceae, such as *Palmaria decipiens*, *Gelidiella acerosa*, and *Gracilaria corticata*) [3,7,19,21,23,25,26,27,28,29,30,31,32].

Marine algae are rich in bioactive compounds such as carbohydrates, proteins, minerals, polyunsaturated fatty acids (PUFAs), fatty acids (FAs), amines, amides, antioxidants (e.g., polyphenols, tocopherols), and pigments such as carotenoids, chlorophylls, carotene, xanthophylls, and phycobilins (phycocyanin (PC), phycoerythrin (PE)), which serve as stabilizing/capping and reducing agents for the synthesis of thermodynamically stable NPs [3,7,21,24,27,28]. It is important to mention that the virtue of biological moieties is influenced by (i) biotic (i.e., type of algal species) and (ii) abiotic factors (e.g., nutrient availability (e.g., nitrogen (N), phosphorous (P), potassium (K)), temperature, pH, salinity, inorganic carbon (C), oxygen (O_2_), light intensity, and carbon dioxide (CO_2_)), as well as by (iii) the dynamic algal-associated halobionts [3,21,24,28]. Most of these factors can have an impact on the production of algae and their metabolites (e.g., primary and secondary), which may subsequently affect the stability, size, and shape of NPs [7,21,24,33,34]. However, for the complete incorporation of these biomolecules in NPs—as food preservatives, prebiotics, antibiofilms, antifouling, antibiotics, or coating (in active packaging)—it is essential to achieve easy, profitable, and sustainable recovery methods [7,24,31,35]. To achieve this purpose, novel solid-liquid, and liquid-liquid extraction techniques (e.g., supercritical, high pressure, microwave, ultrasound, enzymatic, accelerated solvent, and intensity pulsed electric fields extraction) have been studied [7,33,35,36].

NPs synthesized using algae are of two types, (i) organic NPs (e.g., poly-ε-lysine, chitosan, cationic quaternary polyelectrolytes, and quaternary ammonium compounds), and (ii) inorganic/metallic NPs (e.g., Ag, Au, Pt, Pd, Cu, ZnO, TiO_2_, γ-Fe_3_O_4_, and CdS) [3,7,15,21,23]. They are synthesized using two routes of biological extracts, namely (i) extracellular (i.e., NPs synthesized outside the cell, mainly supported by the exudates of cell metabolism comprising metabolites, ions, pigments, lipids, microbial by-products such as hormones and antioxidants, various enzymes, and non-protein entities such as DNA and RNA) and/or (ii) intracellular (i.e., NPs synthesized inside the cell, mainly supported by NADPH or NADPH-dependent reductase originated in metabolic pathways such as photosynthesis, respiration, and nitrogen fixation) [7,15,21,23]. The synthesis of NPs was initially intracellular before its switch to the extracellular mode of synthesis [7].

Among all algal-mediated NPs, metallic NPs (MNPs) are more potent due to their unique optical and electronic properties and biocompatible nature, which has increased their usage in the biomedical field [7,21,23,37]. Their inert nature, low toxicity, and small size (increasing their cell penetration) make them potent candidates for safer and targeted theranostic applications (e.g., drug/gene delivery, gene delivery, immunoassays, tissue repair, laser-assisted therapy (e.g., photodynamic therapy (PDT) and photothermal therapy (PTT)), and/or imaging modalities (e.g., magnetic resonance imaging [MRI], positron emission tomography (PET), biosensing, and cancer chemotherapy)) [1,7,15,21,25].

This article reviewed the literature on marine algae and their theranostic active compounds with emphasis on the qualitative and quantitative analysis of phycocompounds. Biotic (e.g., algal-based halobionts) and abiotic factors (e.g., environmental stress factors on algae) and their impact on the production of algae-derived biomolecules are also highlighted. Further, we present and discuss the potential of algal-based lead nanoencapsulated compounds and their potential for translational medicine, with special emphasis on neurodegenerative diseases and diabetes.

## 2. Algal-Sourced Compounds of Medical Interest

Over the past few decades, marine algae have attracted much interest as potentially renewable resources. There are approximately 8000 different classes of species of marine algae that have been identified in the world [24,27,38,39]. Seaweeds are an excellent source of primary metabolites (e.g., polysaccharides, proteins, amino acids, dietary fiber, essential FAs) and secondary metabolites (e.g., pigments, phytosterols, polyphenols, terpenoids, carotenoids, tocopherols, minerals, and vitamins), which are known to exert cytostatic, anti-viral, anti-helminthic, anti-fungal, and anti-bacterial activities [7,33,35,40,41,42].

Based on the mechanistic differences, physiologically active substances present in marine algae are classified into two types [38,43]: (i) non-absorbed high-molecular materials and (ii) absorbed low-molecular materials, which affect the maintenance of human homeostasis directly. Currently, algal substances are used in fresh and processed foods and have gained importance in nutritional sciences, with promising pharmacological applications as antioxidant, anti-inflammatory, anti-proliferative, anti-thrombotic, anti-coagulant, anti-hypertensive, anti-diabetic, and cardio-protection properties [35,42,44]. Edible algae are therefore used as a food additive/supplement all around the world in the preparation of salads, soups, and low-calorie foods, and represent a regular meal in Japan, Korea, the USA, France, and Chile [38,39,40]. Lately, many clinically viable and commercially available novel drugs with antitumor, anti-infective, anti-diabetic, and neuroprotective formulations from macroalgal biocompounds have emerged as a rising pharmacological field [15,38,42,45,46,47]. Moreover, seaweed biocompounds have revealed their anti-diabetic and neuroprotective effects through various research studies aiming at the prevention of diabetic and neurological disorders (neurodegeneration) and the reduction of oxidative stress in the central nervous system (CNS). The field of seaweed-based anti-diabetic and neuroprotective compounds, however, is still in its infancy, requiring further discoveries and investigations.

### 2.1. Fatty Acid Content

Lipids in seaweeds are present in relatively low contents (i.e., 1–5% of dry weight), and these lipids consist of essential FAs and functional lipid fractions such as PUFAs (i.e., 25% and 60% of total lipids), phytosterols, glycolipids, phospholipids, and fat-soluble vitamins (carotenoids, vitamin A, D, E, and K) [24,48,49].

The most predominant PUFAs occur in the form of omega-3 (eicosapentaenoic acid (EPA; C20:5n−3), docosahexanoic acid (DHA; C22:6n−3), stearidonic acid (SDA; C18:4n−3), α-linolenic acid (LA; C18:3n−3)) and omega-6 (arachidonic acid (AA; C20:4n−6), α-linoleic acid (ALA; C18:2n−6), γ-linoleic (GLA; C18:3n−6)) [24,48]. Essential FAs are nutraceuticals added to dietary supplements or consumed as part of a balanced diet [33]. In 2004, the Food and Drug Administration (FDA) claimed that food containing PUFA omega-3 compounds are pharmacologically important, providing multiple health benefits through their ability to (i) regulate membrane fluidity, blood pressure, and blood clotting; (ii) reduce the risk of cardiovascular diseases (CVD), osteoporosis, and diabetes; and (iii) correct the development and functioning of the brain and nervous system [50,51]. Marine algae such as *Isochrysis galbana*, *Ulva fasciata*, *Laurencia papillosa*, *Gracilaria salicornia*, *Dictyota fasciola*, *Taonia atomaria*, *Chaetoceros*, *Tetraselmis*, *Thalassiosira*, and *Nannochloropsis* are known to produce high amount of PUFAs (ALA, GLA, LA, SDA, AA, and EPA) [15,48]. Furthermore, Peng et al. claimed that green seaweeds like *Ulva pertusa* predominantly contain hexadecatetraenoic, oleic, and palmitic acids [51,52]. EPA, DHA, monounsaturated FAs (C12:1 (lauroleic acid), C14:1 (myristoleic acid), C16:1 (palmitoleic acid), C17:1 (cis-10-heptadecenoic acid), and C18:1 (oleic acid)) are dominant in *Undaria pinnatifida* [48].

More than 200 types of phytosterols (662–2320 mg/g dry weight) have been found in marine algae. Brown algae such as *Agarum cribosum, Undaria pinnatifida*, and *Laminaria japonica* contain major phytosterols derivatives (e.g., fucosterol, which represents 83–97% of the total phytosterol content) [53,54,55,56].

Phospholipids in seaweed vary between 10 and 20% of the total lipids, are more resistant to oxidation (rancidity), and display a high amount of FAs, such as EPA and DHA [40,57].

Glycolipids are present in more than 50% of algal content and are characterized by high n−3 PUFAs compounds (e.g., monogalactosyldiacylglycerides, digalactosyldiacylglycerides, and sulfoquinovosyldiacylglycerides) [33].

Carotenoids are diverse and widespread lipophilic colored compounds in nature, consisting of astaxanthin, β-carotene, lutein, lycopene, and canthaxanthin [13,40]. Moreover, these characteristics give algal lipids better bioavailability and a spectrum of health benefits for humans and animals [15].

### 2.2. Protein Content

Proteins are biological macromolecules present in algae in single (amino acids) or conjugated (heteroproteins such as phycobiliproteins and glycoproteins) forms, and represent 20% and 67%, respectively [57,58]. The highest protein content (i.e., 10–47% of dry weight) was found in edible green (e.g., *Caulerpa lentillifera*) and red seaweeds (e.g., *Eucheuma cottonii*), compared to brown seaweeds (5–24%) (e.g., *Sargassum polycystum*) [51,57,58]. Mohamed et al. reported that most seaweed proteins contain all the essential amino acids at levels close to that recommended by Food and Agriculture Organization (FAO)/World Health Organization (WHO) [59]. Moreover, *Rhizoclonium riparium*, *Enteromorpha intestinalis*, *Lola capillaris*, *Ulva lactuca*, *Dictyota caylinica*, *Catenella repens*, *Polysiphonia mollis*, *Gelidiella acerosa*, *Capsosiphon fulvescens*, *Ulva prolifera*, *Porphyra* sp., *Osmundea pinnatifida*, *Pterocladium capillacea*, *Sphaerococcus coronopifolius*, *Gelidium microdon*, *Cystoseira abies-marina*, *Fucus spiralis*, and *Ulva compressa* have significant levels of proteins [57,58,59]. Proteins display anti-inflammatory, antioxidant, anti-tumor, anti-aging, and protective activity and are therefore beneficial for the prevention and treatment of neurodegenerative diseases, cancers, gastric ulcers, DNA replication, response to stimuli, transport of molecules, and catalysis of biochemical reactions [40,59,60].

Besides, amino acids are applied as natural moisturizing agents to hair and skin, and are therefore beneficial in functional pharmaceuticals, nutraceuticals, and cosmeceuticals [31]. Macroalgal species like *Chlorella* sp., *Dunaliella salina*, *Aphanizomenon flos-aquae*, *Dunaliella tertiolecta,* and *Spirulina plantensis* are widely used as human food sources because of their rich protein content and high nutritive value [42]. Some species of algae are good sources of endogenous (e.g., glutamic acid, aspartic acid, threonine, proline, serine, and glycine) and exogenous (e.g., phenylalanine, histidine, isoleucine, leucine, lysine, methionine, threonine, tryptophan, and valine) amino acids [57,60]. *Ulva australis* contains histidine and taurine, *Ulva* spp. contains aspartic and glutamic acid (26–32% of the total amino acid), and *Palmaria palmata* (Dulse) and *Himanthalia elongata* (sea spaghetti) contain high concentrations of serine, alanine, and glutamic acid, while *Sargassum vulgare* contains a high level of methionine [58,59]. Moreover, mycosporine-like amino acids (MAAs) have been detected in diverse organisms and especially in Rhodophyta; *Chondrus crispus*, *Palmaria palmata*, *Gelidium* spp., *Porphyra/Pyropia* spp., *Gracilaria cornea*, *Asparagopsis armata*, *Solieria chordalis*, *Grateloupia lanceola*, and *Curdiea racovitzae* [60]. MAAs, produced directly or indirectly in algae, can absorb solar energy, and protect marine organisms when exposed to high ultraviolet (UV) radiation [31,61]. Besides, these algal species can be potentially used in cosmetics and toiletries as activators of cell proliferation and UV protectors [31,60,61].

Phycobiliproteins are composed of a protein covalently linked to chromophores called phycobilins (i.e., PC and PE) [57,58]. These water-soluble proteins are good antioxidants and can be used as a natural food colorant [15]. PC, a blue-colored phycobiliprotein produced essentially from the cyanobacteria *Arthrospira* spp., and PE (pink-colored protein pigment) produced by the cyanobacteria *Lyngbya* spp. showed anticancer properties against A549 lung cancer cells [33,40].

Glycoproteins are another type of protein present in marine algae that consist of proteins bound to carbohydrates. About 36.24% of the glycoproteins consist of rhamnose, galactose, glucose, and mannose, with a mole ratio of 38:30:26:6 [15,60].

### 2.3. Carbohydrate Content

Polysaccharides represent 76% of the algal dry weight [15]. These are the major constituents in the cell wall structure of algae, and play important physiological functions [40,41,57]. The algal polysaccharides (e.g., fucan, fucoidans, galactan sulfate, carrageenans, xylomannan sulphate, sodium alginate, fucoxanthin, porphyrin, and alginic acid) found in the cell wall vary with the algae genera and species and can be broadly grouped into sulfated and non-sulfated [40,62,63].

Different amounts of sulfated polysaccharides are found in Chlorophyta (e.g., fucoidans, agar, ulvans, and carrageenans), Phaeophyta (e.g., laminaran, alginate, and fucan), and Rhodophyta (e.g., agar and carrageenans) [41,63]. Minor sulfated polysaccharides such as fucoidans, xylans, and ulvans are found in brown, red, and green seaweeds, respectively [35].

Sulfated polysaccharides extracted from the intercellular space and the fibrillar wall of green seaweeds account for 9 to 36% of algal dry mass in *Ulva* spp. [57]. *Chlorella ellipsoidea* showed several health benefits, such as the capacity to lower blood sugar levels, increase hemoglobin concentration, and act as hepatoprotective and hypocholesterolemic agents. Several food products such as powdered green tea, soups, noodles, bread and rolls, cookies, ice cream, and soy sauce now have been developed with the use of *Chlorella* sp., in which the most important substance is β-1,3-glucan, which is an active immunostimulator, a free radical scavenger, and a reducer of blood lipids [35,60].

Carrageenans are major polysaccharides of the red algal cell wall, and consist of three general forms classified according to the degree of sulphation: kappa, lambda, and iota [33]. Carrageenans, as well as galactan and xylomannan sulphates found in red seaweeds, exert good antiviral properties on the formation of formally similar complexes that block the interaction of the viruses with the cells [64]. Carrageenans obtained from *Hypnea* spp. (but also from the green alga *Ulva lactuca*) exhibit antiviral and antioxidant properties and significant hypocholesterolemic activities by reducing cholesterol and sodium absorption while enhancing potassium absorption [57].

Agar is a mixture of two polysaccharides, namely agarose and agaropectin, which are also extracted from red seaweeds found to have similar structural and functional properties as carrageenans [33,63].

Porphyran, a complex sulfated polysaccharide obtained from the red *Porphyra* spp., has been found to exert immunoregulatory, antioxidant, and antitumor activities [35,41,59].

The sulfated polysaccharides like glucuronic acid, galactose, glucose, rhamnose, and arabinose isolated from the microalgae *Spirulina platensis* exhibited antiviral activity, and those isolated from the red algae *Gracilariopsis lemaneiformis* (i.e., 3,6-anhydro-l-galactose and d-galactose) showed high activity against A549 lung cancer cell line [35,50].

Fucoidans polysaccharides, used to develop novel medicines and functional foods, are generally produced by brown algae such as *Sargassum thunbergi*, *Ascophyllum nodosum*, *Viz fucusvesiculosus*, *Laminaria japonica*, *Fucus evanescens*, and *Laminaria cichorioides* [57]. Algae fucoidans possess antioxidant, antiproliferative, antitumor, antiviral, anti-inflammatory, anti-coagulant, anti-peptic, antiadhesive, antithrombotic properties. They also exhibit high anticancer activity against lung cancer and can suppress lung cancer metastasis by inhibiting matrix metalloproteinases (MMPs) and Vascular Endothelial Growth Factor (VEGF) [40,59]. Fucoidans can present a synergistic effect towards the anticancer agents currently in use [63]. Thus, these polysaccharides can be incorporated into or combined with existing conventional medicines to improve their efficacy. Soluble dietary fibers obtained from *Eucheuma cottonii*, *Caulerpa lentilifera*, *Sargassum polycystum*, *Ahnfeltiopsis concinna*, *Gayralia oxysperma*, *Sargassum obtusifolium*, *Chondrus ocellatus*, and *Ulva fasciata* were shown to reduce blood cholesterol levels and deter metabolic syndrome [40,57].

Alginate (β-d-mannuronic acid, α-l-guluronic acid, d-guluronic, and d-mannuronic) is a commercially available (in acid and salt forms) non-sulfated polysaccharide extracted from the dark brown seaweed *Laminaria digitata* [33,63]. The literature has shown that alginates extracted from brown seaweeds possess a higher nutritional role, and are potentially beneficial in gut health, contributing to water binding, fecal bulking, and decrease of colon transit time, which is a positive factor in preventing colon cancer [41,65]. Moreover, alginates affect the bioabsorption of minerals due to their binding nature, help to maintain body weight and deter overweight and obesity, and reduce hypertension [33,41].

### 2.4. Mineral Content

Seaweeds contain significant amounts of essential minerals, including macroelements (e.g., Na, P, K, calcium (Ca), and magnesium (Mg)) and trace elements (e.g., iron (Fe), zinc (Zn), manganese (Mn), and Cu), due to their marine habitat [38,60]. For instance, the green algae *Ulva clathrata* in México contains a total mineral content of 49.6% of dry matter [57].

Minerals, along with cell surface polysaccharides (e.g., agar, carrageenans, alginic acid, alginate, and cellulose), play an important role in building human tissues and regulating vital reactions as cofactors of many metalloenzymes [40,60]. Hence, seaweeds are an important source of minerals, and are regarded as beneficial functional foods (i.e., food supplements) after daily intake [42]. It is important to mention that the mineral content in brown algae is higher than in red algae [38].

Most edible seaweeds contain relatively higher Na and Ca concentration levels compared to that of terrestrial foods (e.g., apples, oranges, carrots, and potatoes). Intake of low Na:K ratios helps to reduce the incidence of hypertension, and algae usually contain Na:K ratios below 1:5 [57,60]. Besides, minerals like Fe and Cu are present in seaweeds at higher concentration levels than in meats and spinach [57]. Moreover, Cu, iodine (I), Mg, Zn, and Fe are abundant in seaweeds. Iodine is an antioxidant, anti-goiter, anticancer agent, and an important nutrient in metabolic regulation found in several forms (e.g., I^−^, I_2_, IO_2_^−^). However, consumption of very large amounts of I could induce some undesirable effects [57,59].

Arsenic (As) is among the trace elements present in algae that can display poisonous health effects [57]. Nevertheless, further analysis of speciation indicates that the type of As is important in assessing toxicity, and the levels of heavy metals remain normally below food safety limits in most marine algae [48].

Therefore, edible seaweeds could be used as a regular food or as a food supplement to help meet the recommended daily intake of some macrominerals and trace elements [48,60].

### 2.5. Vitamin Content

Vitamins are organic compounds that contribute to essential micronutrients in many biological activities as coenzymes or precursors (e.g., vitamins B6/pyridoxine, B12/cobalamin, and B9/folic acid) and as a part of the antioxidative defense system (e.g., vitamin C/ascorbic acid, carotenoid, and vitamin E/tocopherol) [51,57].

Seaweeds are excellent sources of water (B1/thiamine, B2/riboflavin, B3/niacin, B5/pantothenic acid, B6, B9, B12, C, H/biotin) and fat-soluble vitamins (A/retinoic acid, D, E (which includes α-tocopherol (5,7,8-trimethyltocol), β-tocopherol (5,8-dimethyltocol), γ-tocopherol (7,8-dimethyltocol), and δ-tocopherol (8-methyltocol)), and K) with antioxidant properties [38,48,57,60].

Studies suggest that eating *Spirulina*, which is rich in provitamin A and vitamin B12, increases *Lactobacillus* spp. in the gut and facilitates more efficient absorption of vitamin B1, among many others [38].

Water-soluble vitamins, such as vitamin C, are present in large amounts in *Ulva lactuca*, *Eucheuma cottonii*, *Caulerpa lentillifera*, *Sargassum polycstum*, and *Gracilaria* spp., and help in inhibiting low-density lipoproteins (LDL) oxidation and the formation of thrombosis/atherosclerosis [57]. A relatively high level of dried β-carotene (e.g., 197.9 mg/g in *Codium fragile* and 113.7 mg/g in *Gracilaria chilensis* sp.) was found in red algae compared to other vegetables (e.g., 17.4 mg/g in *Macrocystis pyrifera*) [42,48], while brown seaweeds (e.g., *Undaria pinnatifida*) contain higher levels of a-tocopherol/vitamin E (99% of the total vitamins) compared to green and red seaweeds [60].

The main fat-soluble vitamins (A and E) increase the production of nitric oxide (NO) and nitric oxide synthase (NOS) activity, thereby helping to prevent CVDs [51,57]. Besides, vitamin E exerts an antioxidant activity, which is capable of inhibiting the oxidation of LDL [40].

### 2.6. Pigments

Natural pigments are important for photosynthetic algae metabolism, and based on their pigment contents, macroalgae are classified into three basic groups: Chlorophyceae (green algae), Phaeophyceae (brown algae), and Rhodophyceae (red algae) [33,38,40,51]. Macroalgae can synthesize three basic classes of natural pigments: (i) chlorophylls, (ii) carotenoids, and (iii) phycobilins [38,50]. Macroalgae rich in chlorophylls a and b appear green, while the greenish-brown color of algae is attributed to the presence of fucoxanthin (carotenoid) and the red color of algae is due to the presence of chlorophylls a, c, and d and phycobilins (i.e., PE and PC) [33,51,60,66].

Chlorophylls are greenish lipid-soluble natural pigments that contain a porphyrin ring. These can be divided into four groups: chlorophyll a, chlorophyll b, chlorophyll c, and chlorophyll d [51].

Carotenoids have recently gained interest and are used for dietary supplements, fortified foods, food dyes, animal feed, pharmaceuticals, and cosmetic products due to their antioxidant properties that help reduce the risk of CVDs, cancers, and ophthalmologic diseases [50]. Carotenoids are lipophilic, linear polyenes, and are usually divided into two classes, which are (i) carotenes (α-, γ-, β-) and lycopenes (when the chain ends with a cyclic group, containing only carbon and hydrogen atoms) and (ii) xanthophylls (e.g., fucoxanthin, violaxanthin, antheraxanthin, zeaxanthin, lutein, neoxanthin) or oxycarotenoids (which have at least one oxygen atom as a hydroxyl group, as an oxy-group, or as a combination of both) [33,38,40]. It has been found that α- and β-carotene, lutein, and zeaxanthin are present in red seaweed; β-carotene, lutein, violaxanthin, neoxanthin, and zeaxanthin are found in green seaweed species; and β-carotene, violaxanthin, pheophytins, and fucoxanthin are found in brown algae [33,38,59]. Fucoxanthin, which belongs to the class of xanthophylls and non-provitamin A carotenoids, is found in *Alaria crassifolia*, *Ascophyllum nodosum*, *Chaetoseros* sp., *Cladosiphon okamuranus*, *Cylindrotheca closterium*, *Cystoseira hakodatensis*, *Ecklonia stolonifera*, *Eisenia bicyclis*, *Fucus serratus*, *Hijikia fusiformis*, *Himanthalia elongata*, *Ishige okamurae*, and *Fucus vesiculosus.* It is more effective against Gram-positive (e.g., *Staphylococcus aureus*, *Streptococcus agalactiae*, *Staphylococcus epidermidis*, *pneumoniae*, *Proteus mirabilis*, *Pseudomonas aeruginosa*, *and Serratia marcescens)* and Gram-negative (e.g., *Acinetobacter lwoffii*, *Escherichia coli*, *Klebsiella oxytoca*, *Klebsiella pneumoniae*, *Proteus mirabilis*, *Pseudomonas aeruginosa*, *and Serratia marcescens*) bacteria [51,53,67].

Phycobiliproteins are water-soluble and natural fluorescent proteins that can be divided into three types: (i) PC (blue pigment), (ii) PE (red pigment), and (iii) allophycocyanins (light-blue pigment), with PE being the most abundant in many red macroalgae species [40,50,66]. Algae such as *Spirulina*, *Botryococcus*, *Chlorella*, *Dunaliella*, *Haematococcus*, and *Nostoc* have been recognized as great sources of phycobiliproteins. A recent study has evaluated that these pigments possess antioxidant, anti-carcinogenic, anti-inflammatory, anti-obesity, anti-angiogenic, and neuroprotective activities [40].

### 2.7. Polyphenols

Polyphenolic compounds are secondary metabolites (i.e., not directly involved in primary processes such as photosynthesis, cell division, or reproduction) of algae, and are characterized by an aromatic ring with one or more hydroxyl groups [33,38,57].

Polyphenols are divided into two groups, called phloroglucinols and phlorotannins. Phloroglucinols contain an aromatic phenyl ring with three hydroxyl groups, while phlorotannins are oligomers or polymers of phloroglucinol with additional halogen or hydroxyl groups [40,51,57]. Phlorotannins can be further subdivided into six groups: (i) phlorethols (aryl-ether linkage); (ii) fucols (aryl-aryl bonds); (iii) fucophlorethols (ether or phenyl linkage); (iv) eckols (dibenzo [1,4] dioxin linkages); (v) fuhalols (ortho-/para-arranged ether bridges containing an additional hydroxyl on one unit); and (vi) carmalols (dibenzodioxin moiety) [33,40].

Green and red algae contain high proportions of bromophenols, phenolic acids, an flavonoids, while brown algae predominantly contain phlorotannins (including bromo-, chloro-, and iodo-) [38]. Several reports have evaluated the effective antibacterial effect of phlorotannins, including from *Ecklonia kurome*, against several food-borne pathogenic bacteria (e.g., methicillin-resistant *Staphylococcus aureus* (MRSA) strains, *Campylobacter* spp., and *Streptococcus pyogenes*) [33,57,60].

## 3. Qualitative and Quantitative Aspects of Algal-Derived Biocompounds

Phytochemical profiling of algal samples by advanced analytical techniques revealed the presence and relative amounts of different phytochemicals, many with important medicinal properties (e.g., antimicrobial, anti-inflammatory, antioxidant) [8,24]. Preliminary qualitative phytochemical analysis was carried out to identify the secondary metabolites such as alkaloids, flavonoids, terpenoids, steroids, tannins, phenols, quinones, glycosides, flavanones, flavonols, steroids, and saponins present in the alcoholic/aqueous extracts of marine algae [68,69,70]. The variation in the antimicrobial and antioxidant activities were due to various parameters at the time the algal samples were collected. These parameters include the (i) presence and relative number of secondary metabolites (of phenolic or free hydroxyl nature) in algae, (ii) method of extraction of the biocompounds and the solvent used in this extraction, (iv) maturity stage of algae, and (v) environmental conditions (e.g., habitats, seasons) [70,71,72].

Qualitative colorimetric methods were used to evaluate the phytocompounds, and among the different procedures, methanolic extracts were found to have the highest reducing power in comparison with other solvents, such as ethanol, chloroform, and acetone [70,73]. However, results remain controversial among different studies and seem to be species-specific [70]. The maximum content of phenolic compounds, such as tannins and flavonoids, has been found in red and brown seaweeds [68]. Hasan et al. showed that *Hypnea musciformis* and *Enteromorpha intestinalis* algae collected from the Bay of Bengal possessed high contents of polyphenols associated with high potential of antimicrobial activity [69].

Other phytochemical screenings of different algal extracts were assessed using standard methods. An FeCl_3_ test for tannins in methanolic extracts was assessed for brown seaweeds (i.e., *Dictyota dichotoma* and *Sargassum wightii*), green seaweeds (i.e., *Cladophora glomerata*, *Ulva lactuca*, and *Ulva reticulata)*, and red seaweeds (i.e., *Jania rubens*, *Corallina mediterranea*, and *Pterocladia capillacea*), and the results revealed that tannins are common phytocompounds in seaweeds [68,70]. These algal species can be used as a drug for gonorrhea and as healing agents, and seem to exert anti-viral, anti-bacterial, and anti-ulcer activities [50,65]. A Mayer test was used to qualitatively identify the contents of alkaloids in *Dictyota dichotoma*, *Jania rubens*, *Cystoseira mediterranea*, and *Pterocladiella capillacea* [68]. These are important as antimicrobial agents to inhibit the growth of both Gram-positive and Gram-negative bacteria [70]. Flavonoids, flavonols, quinones and glycosides, flavanones, saponins, and steroids were evaluated qualitatively using the Shinoda test, NaOH test, foam test, and Liebermann–Burchard test, respectively, in different algal species to analyze their therapeutic values [73,74]. In addition, an NaOH test was employed to detect the higher quantity of coumarins in Rhodophyta species (i.e., *Gracilaria salicornia* and *Mastophora rosea*), which, because of their peculiar physicochemical features, were found to display an anticoagulant activity to treat lymphedema [75]. Moreover, saponins and steroids were analyzed through this method in Chlorophyta species (i.e., *Halimeda cuneata* and *Pseudocodium devriesii*) and Phaeophyta (i.e., *Pelvetia wrightii* and *Dictyota dichotoma*) [68,70].

Quantitative analysis of flavonoids, tannins, and phenolics are usually carried out using aluminum chloride assay, 2,2-azinobis 3-ethylbenzothiazoline-6-sulfonate (ABTS) radical scavenging assay, hydroxyl radical scavenging assay, Fe^2+^ chelation assay, and Folin–Ciocalteu reagent (FCR) methods [69,73,76].

As evoked earlier, marine algae also possess a range of macro- and micro-elements required by humans and animals, such as Ca, Na, Mg, K, P, I, Fe, and Zn [72,77]. Semiquantitative and discriminant analyses were used to calculate different percentages of such elements (e.g., Ca, Mg, Na, and K), even within the same group of seaweeds, to differentiate the type of seaweed according to their quantitative mineral levels [77]. For instance, K is known to be present in high proportions in some Phaeophyta species (e.g., *Padina arborescens*, *Hizikia fusiforme*, and *Sargassum thunbergia*), while Ca was in high proportion in other Phaeophyta species (e.g., *Scytosiphon lomentaria* and *Sargassum tortile*). In addition, Mg was found in relatively high quantities in Chlorophyta (e.g., *Ulva conglobata*, *Ulva pertusa*, and *Enteromorpha compressa*), and Chlorine (Cl) was predominantly found in *Pseudocodium devriesii*, *Gracilaria Salicornia*, and *Mastophora rosea* [72,77,78].

Each algal extract obtained is generally mixed with impurities and consists of one or multiple components; therefore, analysis using separation techniques is very important [74]. Different analytical techniques such as high-performance liquid chromatography (HPLC), gas chromatography (GC), thin-layer chromatography (TLC), mass spectrometry (MS), nuclear magnetic resonance (NMR), and one or more combined techniques, such as high performance liquid chromatography–mass spectrometer (HPLC–MS), gas chromatography–mass spectrometry (GC–MS), and high performance liquid chromatography–diode array detection (HPLC–DAD) were used for the identification of bioactive compounds from algal extracts [8,35,36,63,79,80].

Carotenoids and chlorophylls are the most exploited fraction of algae pigments. Due to the lipid peroxidation ability of carotenoids in tissues, in-vivo studies of different biomass extracts were important [65,80]. Furthermore, the total antioxidant activity of carotenoid extracts has been evaluated by UV–Visible (UV–Vis) spectrophotometric methods and/or enzymatic assays [36]. In addition, carotenoids and chlorophylls were quantified by HPLC–photodiode array (HPLC–PDA), identifying all-trans-zeaxanthin, all-trans-lutein, all-trans-β-carotene, all-trans-α-carotene, chlorophyll-α, chlorophyll-β, pheophytin-α, and hydroxychlorophyll-α in the green microalgae *Chlorella sorokiniana* and *Scenedesmus bijuga* [36,80]. Furthermore, HPLC-PDA-MS/MS, HPLC equipped with UV detectors, and MS/MS were used for identification and/or quantification of the carotenoids from algal biomass spectrometry [19,79,81]. Liquid chromatography–mass spectrometry (LC–MS) coupled with PDA and MS showed a high sensitivity for carotenoids and carotenoid esters detection [19]. To investigate antioxidant and anti-cancer properties, the analysis of carotenoids (e.g., β-carotene) has been performed by HPLC–UV/Vis or HPLC–DAD [79,81]. Moreover, for liquid-liquid extracts (analysis done by dissolving the dry extract in the compatible solvents) and the identification of compounds (e.g., astaxanthin, canthaxanthin), HPLC–DAD represents a powerful technique [51].

HPLC is the most sensitive method and is extensively used to separately identify a wide range of compounds like flavonoids and lipids [10,36,81]. Thus, to obtain an adequate measure of the antioxidant potential of individual molecules, pre-column reaction with 2,2-diphenyl-1-picrylhydrazyl (DPPH) radical with ultra-HPLC (UHPLC) separation was used [62,82,83]. Thereby, isoflavonoids, a class of flavonoids, can be structurally distinguished from other flavonoids using HPLC. Isoflavonoids present in brown (e.g., *Undaria pinnatifida, Sargassum muticum*, and *Sargassum vulgare*) and red (e.g., *Hypnea spinella, Halopytis incurvus, Chondrus crispus*, and *Porphyra* sp.) seaweed species were analyzed using modified methodologies of UHPLC–MS/MS [73,81]. In addition, the DPPH free radical scavenging method in cooperation with UHPLC–PDA analysis revealed the presence of two radical scavenging xanthophyll fragments, namely diadinoxanthin and diatoxanthin [84]. Furthermore, HPLC was found to be an alternative method for lipid analysis because it can potentially resolve all the various classes of lipids in crude lipid extracts [81]. Furthermore, HPLC–MS can be used to obtain a more detailed picture of lipid species within each class [79,81]. When using HPLC, sample pretreatment is important; therefore, methanol was used for dissolving the residue, while fat-soluble impurities were extracted with hexane [85]. In some cases, normal phase HPLC coupled in parallel to an evaporative light-scattering detector (ESLD) and quadrupole MS was used to detect a large amount of saturated hydrocarbon in crude lipid extracts [19,81].

In most cases, especially for analytical research and the development of nutraceuticals, it is necessary to evaluate the suitability of the analytical techniques. Algal lipid quantification is generally carried out based on indirect methods, such as Nile red fluorescence or related dye-partition assays, gravimetric measurement of crude lipid extracts, or GC analysis of lipid-derived fatty acid methyl ester (FAME) [51,81,85]. Numerous anomalies can affect neutral lipid quantification, including distortions due to β-carotene, complex kinetics of the fluorescent signal, and issues with sensitivity or specificity. Nile red fluorescence is visibly specific for lipid droplets, and is used as one of the most popular methods of algal lipid analysis [81].

GC/MS and NMR techniques are also used for lipid analysis [81]. GC is a popular method used on its own and/or in combination with various detection techniques such as PDA, UV, MS, MS/MS, HPLC, electron capture detector (ECD), and flame ionization detector (FID) [79]. With GC analysis, acyl constituents and FAME, derived from both neutral and polar lipids, can be selectively analyzed in each lipid extract [85]. Algal-derived FAs, as methyl or ethyl esters, could be then analyzed by LC–MS and/or GC–FID [85]. Moreover, post-methylated lipid analyses can be carried out using GC–MS. Reversed-Phase HPLC (RP–HPLC) was a widely applied analysis method, but this technique fails to separate highly polar compounds from the less polar ones [36]. Therefore, capillary electrophoresis (CE) using DAD (CE–DAD), which shows shorter application time, higher efficiency, and selectivity, is used as a substitute method to RP–HPLC for fast SFE extracts characterization [36].

NMR, MS, HPLC–MS, HPLC–UV–MS, and GC–MS have been applied to perform a pharmaceutical-grade analysis of biocompounds. For terpenes, GC–MS or NMR were found to be applied for structural determination. GC coupled to an electrospray ionization (GC–ESI) and GC–MS analyses are very selective for identification of heat-labile components (e.g., volatile materials, hydrocarbons, and FAs) in phytoextracts [36,79]. 1D- and 2D-NMR, MS/MS, HPLC, and chiral GC–MS analyses are preferred for structure evaluation [63,84]. Proton NMR (^1^H NMR) spectroscopy has gained attention as a good analytical tool for structural analysis of polysaccharides (including determination of monosaccharide constituents, partial depolymerization by reductive hydrolysis, identification of disaccharide repeating units) and sequence analysis by enzymatic degradation due to its advantages of simple calibration, easy application, and fast optimization of the experiment [36,63,79]. However, this technique was only suggested for chemical identification and not quantification, due to possible structural irregularities, which could lead to misleading and complex signals. The linkage positions of carbohydrates and the linking relationships are determined concomitantly with heteronuclear single-quantum correlation spectroscopy (HSQC) and heteronuclear multiple bond correlation spectroscopy (HMBC) [36,84,86]. Globally, hydrocarbons characterization is mainly done by GC/MS and NMR [87].

Thermogravimetric analysis (TGA), differential scanning calorimetry (DSC), and dynamic mechanical analysis (DMA) were used to analyze thermal properties of polysaccharides, lipids (from supercritical extracts), and algal proteins, which can be quantified by determining the nitrogen content using Kjedhal analysis [36,86].

Infrared (IR) spectrometry is a common analysis technique used to identify functional groups present in algal extracts [79]. Thereby, glycoprotein structural details (e.g., sugars attached to the protein via (1→4)-linked β-galactose residues and β-linked glucose residues) have been elucidated using Fourier-transform infrared (FTIR) and NMR spectra [54,80]. Furthermore, glycoproteins obtained from *Codium decorticatum* were purified and characterized using HPLC, IR, NMR, and Circular Dichroism (CD) [15]. Generally, IR-KBr plate (mixing the powder sample with potassium bromide (KBr) and then pressing it into a disc mode) helped to identify algae’s (e.g., Ulvan’s) chemical components [36,86]. Further, attenuated total reflectance-FTIR (ATR-FTIR) and Raman spectroscopy techniques are used to identify agar and other polysaccharides sources of seaweeds [63]. Spirulina is an important edible alga with increasing commercial interest, and a faster and more highly efficient analytical platform was introduced to qualitatively and quantitatively characterize *Spirulina* pigments in different dietary supplements [87]. Thereby, analysis of the *Spirulina* pigment fraction was possible through a highly complex and developed analytical strategy, consisting of Fourier-transform ion cyclotron (FT–ICR) in both direct infusion (DIMS) mode or coupled with UHPLC. This strategy was used to accurately identify and overcome failures of conventional LC–MS-based methods (e.g., low separation efficiency, long analysis time, and low mass accuracy) [79,87].

TLC can be employed to elute extracts of chlorophyll α and multiple carotenoids, such as β-carotene, oscillaxanthin, zeaxanthin, β-cryptoxanthin, echinenone, and myxoxanthophyll [87]. The TLC method evaluates both quantitatively and qualitatively extracted algal components (e.g., hydrocarbons) among different solvents (mobile phases such as acetic acid/hexane/acetone/diethylamine/diethyl ether) and temperatures [36,87].

Several chromatographic methods, such as TLC, HPLC, GC, high-performance anion-exchange chromatography-pulsed amperometric detector (HPAEC–PAD), and CE, have been used for the separation and selective analysis of agaro-oligosaccharides (AOS) [35,84].

ESI and matrix-assisted laser desorption/ionization (MALDI) have advanced the structural analysis of AOS and carrageenan oligosaccharides (COS). Different fragmentation patterns were obtained by ESI-tandem MS due to sulfation substitution allowing researchers to selectively detect COS among other polysaccharides [35,63]. Thereby, detailed oligosaccharide information, such as accurate molecular weight, chain length distribution, fragments information, monosaccharide compositions, linkages, and location of various modifications, has been identified [35]. Recently, MS has been used as a powerful detection tool for elucidating the oligosaccharide structure due to its sensitivity [79,84].

For the quantitative analysis of toxins, LC–MS/MS methods have proven their efficiency, although they are limited for multi-component analyses (MCA) [36].

## 4. Influence of Biotic and Abiotic Factors in the Production of Algal Biocompounds

Recent research has focused on improving synthesis and maximizing the production of valuable biocompounds from algae cultures. Most algae are able to synthesize numerous therapeutic compounds such as ash (8.4–43.6%), high fiber (5.3–52.3%), low protein (4.9–37.8%), and small amounts of FAs (0.92–5.2%) [33,43]. Environmental factors, such as nutrient availability (N, P, K, etc.), salinity, temperature, inorganic carbon, oxygen, light intensity, CO_2_, pH of the seawater, age of the fronds, and sampling seasonality [24,88,89], influence the algal growth, the overall yield of biomass, and the synthesis of therapeutic biocompounds such as lipids, proteins, polysaccharides, vitamins, pigments, and minerals [28,75]. Therefore, establishing the reference values for polysaccharides, minerals, and trace elements available in algae remains difficult. Abiotic stress conditions can also have negative effects on marine algal growth [28,48].

The pH of the environment is an important factor affecting algae growth and prevention of contamination by microorganisms or other species [24,65]. The pH control is essential for effective absorption of the components of the culture medium because it directly affects the bioavailability and stability of various chemical elements. Algae species have different pH requirements for the growth and absorption of nutrients, and a change in the pH causes harmful effects for algae cells [88]. For instance, the green microalga *Chlorella vulgaris* can grow in a broad range of pH values; however, optimum growth rate and biomass productivities were observed at pH 9–10 [90].

Moreover, variation of nitrogen consumption by algae affects the metabolic cycles, causing changes in the production of lipids or carbohydrates [65,88]. Peng et al. showed that the growth of the green seaweed *Ulva pertusa* in a high-salinity environment resulted in a high content of total FAs [51].

Light duration and intensity are the major limiting factors that substantially affect the biochemical composition of microalgae and biomass yield [24,88]. Light also negatively affects cell reproduction, impacting algal growth efficiency. Higher light intensities will enhance the photosynthetic rate; besides, the increasing rate is balanced by photorespiration and photoinhibition up to its threshold value, and beyond that, the cell get disrupted [41]. The FAs composition in algae is commonly influenced by different ambient conditions such as light intensity, seawater salinity, and temperature. This is because lipids are the major components of chloroplasts, and the increased light intensity demands greater activity of chloroplasts. Increased levels of most saturated FAs (SFAs) were observed [50,65]. Nevertheless, FAs in the green seawed *Ulva pertusa* increased with increasing light, while the contents of almost all FAs in the brown seaweed *Sargassum piluliferum* were decreased [51], suggesting that light-induced production of FAs depends on the class of algae. The studies also showed that the incident light intensity on the algae alters its antioxidant potential. Thereby, the application of blue light in the culture of the cyanobacterium *Spirulina fusiformis* alters the sequence of amino acids with cysteine repeats, leading to increased antioxidant capacity [65].

Temperature is identified as another important factor impacting the growth of algae, production of biomass (like proteins, lipids, and phenolic compounds), and biochemical processes [7,24,88,91]. Each algal species has its optimal growth temperature, but the optimum temperature range for most algal species was identified between 35 °C and 37 °C [51]. For instance, the cyanobacterium *Synechococcus leopoliensis* produced a maximum concentration of bioactive compounds at a temperature of 35 °C and pH 8 [65]. A decrease in the temperature beyond the optimal level affects photosynthesis by reducing carbon assimilation activity that will retard or stop algae growth and activity [91]. Conversely, an increase in the temperature beyond the optimal level reduces photosynthesis, cell size, and respiration, subsequently inactivating the photosynthetic proteins and disturbing the balance of energy in the cell [65,71,92]. It was reported that FAs levels are influenced by low temperatures in different species [51]. Moreover, photosynthesis and respiration are temperature-dependent, and the specific growth rate of the microalgae is directly correlated with the gross rate of CO_2_ fixation/O_2_ production and the respiration rate [93]. At the end of summer, the growth of the brown seaweed *Undaria pinnatifida* (wakame) reached a maximal size of up to 4 m with average water temperatures of between 10.2 °C and 11.4 °C [94]. The total FAs content in the brown seaweed *Egregia menziesii* reached its highest content in spring (13.3 mg/g of dry weight) and lowest in summer (6.3 mg/g of dry weight) [95]. FAs in algal cell membranes are temperature-sensitive, and at low temperatures, the level of unsaturated FAs (UFAs) increases by increasing the levels of polar lipids [96]. This causes melting points depression and maintain lipids in a liquid state for normal protoplasmic viscosity. Moreover, saturated FAs (SFAs) compositions are also temperature-sensitive, and their level can also be controlled by temperature [49,96]. The Rhodophyta *Palmaria palmata* had higher levels of EPA at 11 °C and AA at 15 °C, and the Phaeophyta *Saccharina japonica* reached its highest level of (n-6) PUFA content during the high-temperature season, whereas (n−3) PUFA levels were the highest during the cold months [94].

Nutritional requirements vary and depend on algal species. Nutrient absorption is species-dependent; red and green species are rich in carbohydrates, whereas brown algae are rich in iodine and soluble fiber [48]. However, the basic requirements are the same for all species. Indeed, N, P, and C (CH1.7 O0.4 N0.15 P0.0094) form the backbone of algae [65,88]. These nutrients are involved in the growth of algae through various enzymatic reactions and, thus, can alter their biosynthetic pathway to form and accumulate a bioproduct of interest [65]. There are different mechanisms involved in nutrients uptake by algae. Gases such as CO_2_, O_2_, and NH_3_ and uncharged molecules are taken by passive diffusion along the concentration gradient, whereas inorganic elements, like nitrogen and phosphorous, are taken up by active transportation [72].

Under similar environmental conditions, geographical origins, and harvesting times, mineral and trace element concentrations vary tremendously among the different families/genera of algae [97]. The amount and composition of polysaccharides in algal cell walls may differ in their rates of biosorption of minerals and trace elements [63,98]. Minerals and polysaccharides found in the cell wall vary with the microalgae genera and species [38,51]. The green alga *Ulva lactuca* as well as the red algae *Jania rubens* and *Pterocladia capillacea* collected from the western coast of Alexandria (Egypt), particularly from spring to autumn, were subjected to analysis of their protein and carbohydrate contents [96]. The highest protein and carbohydrate contents were found in *P. capillacea*, while *U. lactuca* contained more lipids and proline than the other two species. Moreover, *J. rubens* demonstrated higher levels of total FAs, but *P. capillacea* displayed higher levels of SFAs, mainly because of the presence of palmitic acid. PUFAs levels were the highest in *J. rubens* due to dominance of DHA. In another research, algal species collected from northern and southern Portugal, in summer, show seasonal reproductive phenology and different polysaccharides concentrations in algal cell walls [99]. It has been found that temporal changes occurred mostly within the tip tissues and less within the basal blades, which could lead to a prominent change in nutraceuticals of marine algae.

## 5. From Basic Research to Translational Nanomedicine: Advancements and Prospects

### 5.1. Algal-Derived Compounds and Derived Nanotheranostics for Diabetes

Diabetes mellitus (DM) is a fast-growing non-transmissible disease worldwide. In recent WHO and Internal Diabetes Federation (IDF) statistical reports, the number of diabetic patients has been estimated to exceed 382 million in 2013, with a potential increase to approximately 600 million cases globally by 2035 [100].

DM is currently recognized as a major global health problem that affects young and adult individuals, resulting in increased morbidity and mortality. According to the WHO and the American Diabetes Association (ADA), DM is defined as an endocrine metabolic disease characterized by prolonged and persistent hyperglycemia caused by either inadequate insulin production or insulin action or a combination of both [101,102].

According to the WHO and ADA, there are two main types of DM, namely Type 1 DM (T1DM) and Type 2 DM (T2DM). T1DM (or insulin-dependent DM) is a complex multifactorial autoimmune disease resulting in immune-mediated pancreatic β-cell damage and complete lack of insulin production because of a complicated interaction process between genetic causes, environmental factors, such as viruses and infections, and the host immune system [103,104]. Although the pathological basis of T1DM is not entirely understood, as many genetic and environmental factors are involved in triggering the disease, some studies have revealed that T1DM is an autoimmune disease caused by a cascade of autoimmune responses against pancreatic β-cells resulting from the interaction between genetic and environmental factors or a combination of both. This interaction leads to the activation of the adaptive immune system via both types of T-lymphocytes (CD4+ and CD8+), resulting in systemic lymphocytic infiltration of pancreatic β-cells [105]. The infiltration of pancreatic β-cells causes insulitis, which in turn leads to an increase in the expression of key inflammatory cytokines such as interleukin 1β (IL-1 β), tumor necrosis factor-α (TNF-α), and interferon-γ (INF-γ), which induce apoptotic events and complete destruction of pancreatic β-cells and diabetes [105,106,107].

T2DM is a multifactorial heterogenic disease characterized by peripheral insulin resistance or insufficient insulin production by pancreatic β-cells or both, resulting in increased hepatic glucose production [101,104,108]. In the early stage of T2DM, the insulin level is abnormally increased, which indicates a cellular signaling impairment rather than an alteration in insulin production, because of reduced peripheral insulin receptor sensitivity and signaling which results in insulin resistance [109,110,111]. T2DM is a complicated genetic disease that involves genetic defects in pancreatic β-cell functioning and insulin signaling. Genome-wide association (GWA) studies have identified more than 40 loci that are linked to the pathogenesis of T2DM, such as KCNJ11, TCF7L2, and KCNQ1, which are associated with pancreatic β-cell functioning, and PPARG, IRS1, IGF1, IGF2, and KLF14, which are concomitant with insulin resistance [109,110,112]. Additionally, several specific types of diabetes have been identified. However, the relative underlying risk for developing DM relies on causal factors [101]. These specific types of diabetes include gestational DM, which is associated with hyperglycemia because of glucose metabolism impairment during pregnancy, which may subsequently increase the likelihood of developing hypoglycemia in the infant and T2DM later in life [101,113]. Genetic defects in pancreatic β-cell functioning (known as maturity-onset diabetes of the young, MODY) is caused by genetic mutation or defect in a single gene in different chromosomes in an autosomal dominant manner leading to insufficient insulin production and hyperglycemia [101,114].

Among marine algae, the brown algae exert effective biologic activities including antidiabetic, anti-inflammatory, cytotoxic, and antioxidant activity, and produce the most important secondary bioactive metabolites such as chlorotanins, fucosterols, fucoidan, alginic acids, and phycoxanthin [25]. Considering that there is also a desire for safe and effective antidiabetic medicinal products for diabetes management, the use of algae-derived compounds can be an asset in the treatment of this pathology. Indeed, when searching for successful anti-diabetic drugs, marine algae remain a promising source with powerful bioactivity [45,115]. The isolation, classification, and pharmacological analysis of unexplored marine algae is anticipated to be useful in the discovery of novel high-biomedical-value antidiabetic compounds. Brown and red algae displayed antidiabetic activity [116]. Most of the research conducted with algal-derived compounds showed a regulation of blood glucose levels, by inhibiting carbohydrate hydrolyzing and protein tyrosine-phosphatase 1B enzymes, sensitivity to insulin, glucose absorption, and other diabetic protective effects [117,118]. Such compounds can be directly extracted from the marine particles present in the algae.

As a proper alternative to hazardous chemistry and physical synthesis, significant attention was paid to the biosynthesis of metal NPs using medicinal plants. Plants are used for their specific metal tolerance and efficient AuNPs output [21,119,120]. A single plant comprises an orchestra of environmentally benign chemical components that serve as ideal instruments for enhanced medicinal applications, including protein, vitamins, enzymes, amino acids, polysaccharides, and organic compounds. It has been stated that the bio-reduction, stabilization, and bio-capping mechanisms for the formation of stable AuNPs and AgNPs include phytocomponents like terpenoids, polysaccharides, polyols, and flavones [21,119]. The inhibitory ability for plant compounds with diabetic objectives, followed by an analysis of the enzyme inhibitor kinetics, the binding of ligand dynamics aided by silico docking studies, reveals the mode and inhibitory activity [119]. Due to the availability of bioactive elements, the antidiabetic capacity of marine algae has been extensively studied in recent years.

Among the bioactive compounds highly present in brown algae, phlorotannins have been identified as a possible source of medication for a variety of human diseases, including diabetes [47,121]. Phlorotannin subgroups are found in numerous brown algal species, which by means of many pathways have essential antidiabetic functions. Overall, in vitro and in vivo (in animal models) assays have reported a potential hypoglycemic effect of marine brown algae through various pathways [121]. Fucosterol that was isolated from the brown alga *Pteris siliquosa* caused a decrease in serum glucose levels and inhibited glycogen degradation in streptozotocin (STZ)-induced diabetic rats [122]. An extract from the brown alga *Pelvetia babingtonii* exhibited potent α-glucosidase inhibitory activity and was effective for suppressing postprandial hyperglycemia [123]. Table 1 shows a summary of brown algal phlorotannins and their possible anti-diabetic effects. Table 2 shows a summary of seaweed-derived compounds and their anti-diabetic effects. In addition to brown algae, there are some red macroalgae that showed antidiabetic activity. Table 3 shows the bromophenols from red algae as algal enzyme inhibitors linked to DM. Eventually, polysaccharides isolated from the green seaweed *U. Lactuca* (sea lettuce) could decrease blood glucose by their potential inhibitory effect on enzymes closely related to starch digestion and absorption in both plasma and the small intestine [124,125]. The ethanolic extract of *Ulva rigida* also decreased blood glucose concentrations in diabetic rats [126,127].

Different algae (e.g., Cyanophyceae, Chlorophyceae, Phaeophyceae, Rhodophyceae) can be considered as candidates in the biosynthesis of AgNPs due to their properties of fast growth, abundant organic content, and high metal accumulation ability [3,17,169]. AgNPs biosynthesized from the marine red macroalga *Halymenia poryphyroides* showed in vitro antidiabetic activity by inhibiting both α-amylase and α–glucosidase enzymes in a dose-dependent manner [169].

To improve the quality of life for patients with insulin-dependent diabetes, several nano-technological approaches have been developed [47,119,169]. They make blood glucose control easier by allowing non-invasive glucose monitoring and insulin administration, primarily by delivering the fragile protein in a safe and targeted formulation through the nasal or oral path [47]. New generations of selective nanoparticle-based drugs are being produced and tailored for specific metabolic conditions (including age-related, disease development), which is a crucial stage [47,119]. Not only in nanomedicine, but also in pharmacology in general, the impact of age-related factors (such as immaturity in very young children, metabolic and physiologic changes in old age) are still understudied, due to the use of inadequate animal models [7]. It should be noted that insulin administered through routes other than subcutaneously has a bioavailability of only 60% [42]. Furthermore, factors such as altered gut permeability, as defined in T1DM, or other metabolic peculiarities, such as insulin resistance in T2DM, may affect the production of novel nanoparticulated drug preparations and be responsible for the failure to convert promising animal results into human therapy [106,109].

Taken together, future insulin production by NPs must consider not only the drug’s requirements, but also the metabolic changes caused by disease or ageing [42]. Furthermore, adequate animal models and approaches to disease prevention are needed.

### 5.2. Algal-Derived Compounds and Nanotheranostics for Neurodegenerative Disorders

Evidence shows that neurodegenerative diseases will become our greatest threats. They are estimated to surpass cancer as the second most common cause of death among the elderly after CVDs by the year 2040 [170], and over 130 million people worldwide will have dementia by 2050 [167].

Neurological disorders are characterized by the region-specific loss of neurons; they include a range of pathological conditions including Alzheimer’s disease (AD), Parkinson’s disease (PD), multiple sclerosis (MS), Huntington’s disease (HD), amyotrophic lateral sclerosis (ALS), and traumatic brain injury (TBI) [171]. AD and PD are the most prevalent neurodegenerative diseases in the elderly.

AD and PD share common pathophysiological traits [172]. Neuropathological studies have reported that AD was associated with multiple factors that contribute to its development, including progressive deterioration of synaptic neurons, oxidative stress, accumulation of Aβ (a component of the amyloid plaques) in the brain, as well as reduction of neurotransmitter acetylcholine (Ach) levels in the hippocampus and cortex of the brain [173]. This form of dementia is characterized by memory loss, behavior disturbances, personality changes, and decline of cognitive abilities [174]. Some important biological pathways engaged with the pathogenesis of PD include the degeneration of dopaminergic neurons in the substantia nigra, intraneuronal aggregation of neurofibrillary tangles (NFT), as well as extracellular aggregation and accumulation of α-synuclein protein of Lewy bodies (LB) [175]. PD predominantly manifests with muscular rigidity, bradykinesia, rest tremor, impairment of dexterous movements, and nonmotor symptoms [176]. However, the molecular mechanisms of neurodegeneration are not fully understood, and the major mechanisms leading to neurodegeneration are multifactorial, triggered by genetic, environmental, and endogenous factors related to aging.

Common underlying pathogenic mechanisms of many neurodegenerative disorders include neuroinflammatory and/or neuroimmune processes, extensive oxidative and/or nitrosative damages caused by the formation of free radicals (e.g., reactive oxygen species (ROS) and reactive nitrogen species (RNS), mitochondrial dysfunction, and synaptic loss leading to cell dysfunction and cell death [177]. Therefore, mechanisms to regulate oxidative stress inflammatory response could prove to have important therapeutic potential for the treatment of neurodegenerative diseases [178].

Despite scientists having made remarkable strides in understanding how neurodegenerative disorders affect the brain during the last few decades, the increasing social, health, and economic burden of these diseases worldwide demands a great attention from the scientific community. One such focus is developing potential bioactive substances with therapeutic potential that can inhibit disease pathogenesis without causing undesirable effects in patients’ health [179]. Currently, the research for inhibitors of dementia and AD continues in many countries around the world. Compared to synthetic therapeutics, use of naturally derived drugs in the treatment process produce well-tolerated therapeutic effects and multi-neuroprotective properties that pose little or no side effects [180]. Furthermore, it has been reported that neurodegenerative disorders such as AD are strongly correlated with poor nutrition. Therefore, improving diet through functional foods, nutraceuticals, and supplements (e.g., edible algae, algal-derived active compounds) could have a potential to be used as a therapeutic intervention in the management of disorders like AD [181]. Natural compounds have been shown to possess anti-inflammatory, antioxidant, and immunomodulatory effects, which are important therapeutics in many neurodegenerative diseases. In this sense, diverse natural products with a wide range of biological activities able to decrease the symptoms and protect against the development of several neurological diseases including AD have gained the attention of the scientific community and the pharmaceutical industry [182].

Undoubtedly, the marine environment represents a major reservoir of bioactive compounds [183]. Among marine organisms, seaweeds have been identified as under-exploited plant resources that provide an excellent choice to explore for applications in the renewable energy, food, pharmaceutical, nutraceutical, and cosmetic industries [29,30]. In addition, due to their diverse primary and secondary metabolites with a wide range of pharmacologically active components and biochemical characteristics, marine microalgal compounds have received much interest as important chemical scaffolds for the discovery of new drugs for the management of some chronic diseases, including neurodegenerative disorders [184]. Further, marine algae are considered as a valuable food source, commonly consumed across parts of Asia. An epidemiological study that compared Japanese and Western diets demonstrated that there is an association between algae consumption and a lower incidence of chronic degenerative diseases [185]. Previous studies have revealed that compounds from marine algae exhibit various biological activities such as antioxidant [186,187], anti-inflammatory [171,186,188], anticoagulant [189], antiviral [190], anti-cancer [187,191], antidiabetic [142], and anti-allergic [192] properties. Furthermore, several studies have demonstrated the role of these algal compounds in neuroprotection [30,174,178,179,193]. A recent review by Rengasamy et al. [194] compiled various bioactive compounds derived from marine algae and their role as enzyme inhibitors, showing great potency for treating multiple diseases, including cancer, diabetes, inflammation, and dementia, among others.

**Figure 1:**
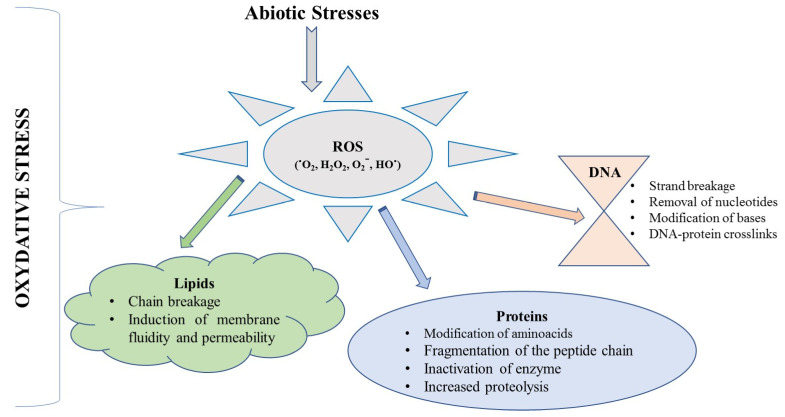
Reactive oxygen species (ROS) induce oxidative damage to lipids, proteins, and DNA. Adapted from [201].

**Figure 2:**
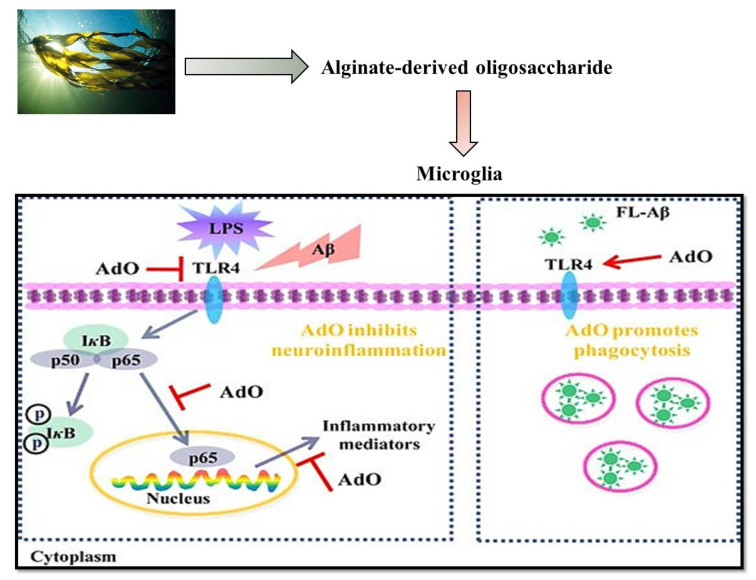
Alginate-derived oligosaccharide inhibits neuroinflammation. Adapted from Ref. [206].

OS has been implicated in the pathogenesis of neurodegenerative diseases including AD, PD, and MS. OS (Figure 1) are induced by imbalanced redox states (pro-oxidant and antioxidant levels) or dysfunction of the antioxidant system leading to excessive generation of ROS [195]. The CNS is more sensitive to oxidative stress compared to other parts of our body. This is because of the high oxygen demand and lipid content of the CNS. Elevated oxidative stress in the CNS leads to lipid peroxidation and DNA and protein damage and eventually triggers excitotoxicity and apoptosis, two main causes of neuronal death [196]. Mitochondrial dysfunctions could also be a result of excessive ROS generation, establishing a vicious cycle of OS [197]. The cell systems that deal with the biochemistry of OS are complex and not well understood. Antioxidants have been shown to have important therapeutic effects as they can protect the CNS against free-radical-induced oxidative damage. However, endogenous antioxidant is always ineffective, and the human body is constantly exposed to damaging environmental factors. Therefore, exogenous antioxidants are vital in diminishing the cumulative effects of oxidative damage [178,187]. Currently, antioxidants are considered essential in the prevention of neurodegenerative diseases and therapy. Therapeutic approaches using compounds that exhibit anti-oxidative properties, for example novel metal-protein attenuating compounds, were shown to slow down the progression and limit the extent of neuronal cell loss in these disorders [198]. Many methods have been employed for the determination of antioxidant activities exerted by marine algae, including lipid peroxide inhibition, free radical scavenging, and singlet oxygen quenching activity [178]. Recent studies on marine algae compounds, such as fucosterol, fucoxanthin, sulfated oligosaccharides, phlorotannins (dieckol), showed neuroprotective effects related to ROS scavenging activities, inhibition of cholinesterases, and protection against β-amyloid aggregation and neuronal damages [29,179,199,200]. For instance, the marine red alga *Neorhodomela aculeata* was able to scavenge DPPH in hydrogen peroxide (H_2_O_2_)-induced lipid peroxidation in rat brain homogenates [200]. ROS scavenging activity was also observed in the green seaweed *Halimeda incrassata* and the red seaweed *Bryothamniom triquetrum* [199].

(Neuro)inflammation has been known to be the main pathophysiological mechanism in neurodegenerative diseases such as AD and PD. To protect the CNS against damages or external pathogenic infections and other threats, acute neuroinflammation acts as a defense mechanism, which is beneficial to restore homeostasis. However, chronic neuroinflammatory processes may lead to cascades of events that cause progressive neuronal damages, such as those observed in AD and PD [202]. Several algal compounds were shown to exhibit anti-inflammatory activities. Algal phenolic compounds (e.g., phenolic acids, flavonoids, phlorotannins, coumarins, lignins, lignans, stilbenes, and their derivatives), as well as other compounds (e.g., lipid derivatives such as PUFAs, polysaccharides such as fucoidans and carrageenans), were shown to exert anti-inflammatory effects [203,204,205]. These algal compounds modulate neuroinflammation by acting at different cellular levels and pathways, such as modulating mitogen-activated protein kinase (MAPK) pathways and nuclear factor kappa-B (NF-kB) activation [171]. Studies on alginate-derived oligosaccharide (AdO) from marine brown algae on lipopolysaccharide (LPS)/β-amyloid (Aβ)-induced neuroinflammation and microglial phagocytosis of Aβ (Figure 2) revealed dual effects of AdO on BV2 microglial cells, where AdO exerted an inhibitory effect on the LPS/Aβ-activated inflammatory response and promoted microglial phagocytosis of Aβ [206].

**Figure 3:**
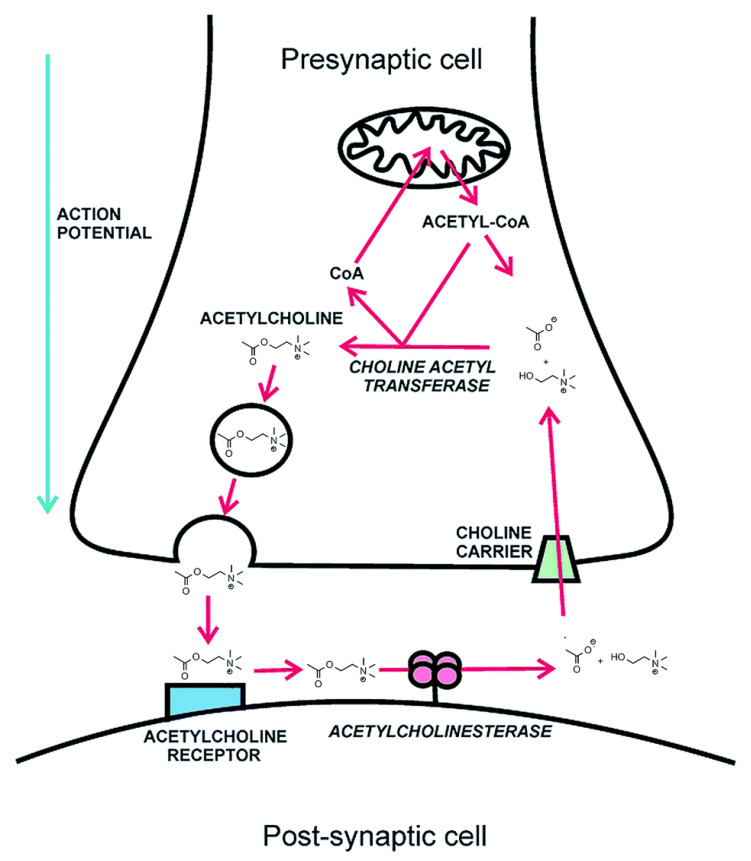
Natural cholinesterase inhibitors from marine algae. Adapted with permission from Ref. [210]. Copyright 2019 the Royal Society of Chemistry.

As evoked earlier, neurodegenerative disorders such as AD are also characterized by a loss of cholinergic function in the CNS and reduced level of the neurotransmitter ACh. The inhibition of AChE enzyme was hypothesized to be the most effective approach to the symptomatic treatment of AD [204]. Apart from increasing ACh levels, cholinesterase inhibitors (ChEi) also prevent Aβ-induced neuronal death by modulating the α-secretase activity (which acts on the amyloid precursor protein), thereby inhibiting β-amyloid aggregation [203]. A list of marine algae has been reported to have significant AChE inhibitory activity that could be effective in neurodegenerative diseases such as AD [193]. Algal compounds (Figure 3) such as dieckol and phlorofucofluoroeckol were shown to possess memory-enhancing and AChE-inhibitory activity [207]. Compounds isolated from extracts of marine green microalgae including *Nannochloropsis oculata*, *Chlorella minutissima*, *Tetraselmis chuii,* and the red microalga *Rhodomonas salina* were shown to inhibit in vitro AChE activity [208]. Deficiency of omega-3 PUFAs has been linked with the early onset of AD, whereas PUFAs have exhibited neuroprotective activity and can improve neurotransmission in cholinergic neurons [30,209].

Taken together, the bioactivities and neuroprotective effects of marine algae are mainly mediated through their capacity to minimize OS, neuroinflammation, and inhibition of the AChE enzyme activity. Table 4 summarizes algal-derived bioactive compounds that could be nanoencapsulated to enhance their effectiveness against neurodegenerative diseases.

## 6. Conclusions

Marine algae remain a largely unexplored reservoir of natural theranostic products (e.g., antioxidants, antimicrobials) that could be further nanoencapsulated to enhance efficiency and safety. Both biotic and abiotic factors can impact the algal production of such compounds. Interestingly, a green approach to NPs synthesis using dead or living algae can serve to nanoencapsulate algal compounds. Phlorotannins and eckol derivatives, bioactive compounds highly present in brown algae, have been identified as possible sources of (adjuvant) medications for diabetes and Alzheimer’s disease, respectively. More research should be conducted toward the development of algal-derived compounds and nanocompounds for treating complex chronic diseases.

## Figures and Tables

**Table 1:** Brown-algae-derived phlorotannins and their diverse anti-diabetic effects. Adapted with permission from [128]. Copyright 2013 Elsevier.

Phlorotannins	Anti-Diabetic Effects	Sources	References
Dieckol	α-Glucosidase inhibitor	*Ecklonia cava*	[129]
	Postprandial-hyperglycemia-lowering		[130]
	PTP 1B inhibition		[131]
	Protective effect against diabetes complication		[132]
Fucodiphloroethol G	α-Glucosidase inhibitor	*E. cava*	[129]
6,6′-Bieckol	α-Glucosidase inhibitor	*E. cava*	[129]
7-Phloroeckol	α-Glucosidase inhibitor	*E. cava*	[129]
	PTP 1B inhibition		[131]
Phlorofucofuroeckol A	α-Glucosidase inhibitor	*E. cava*	[129]
	PTP 1B inhibition		[131]
Phloroglucinol	α-Glucosidase inhibitor	*E. stolonifera*	[131]
	PTP 1B inhibition	*E. bicyclis*	[131]
Dioxinodehydroeckol	α-Glucosidase inhibitor	*E. stolonifera*	[131]
	PTP 1B inhibition	*E. bicyclis*	[131]
Diphlorethohydroxycarmalol	α-Glucosidase inhibition	*Ishige okamurae*	[133]
	Postprandial-hyperglycemia-lowering		[133]
	Protective effect against diabetes complications		[134]
Eckol	α-Glucosidase inhibitor	*E. stolonifera*	[131]
	PTP 1B inhibition	*E. bicyclis*	[131]
Octaphlorethol A	Glucose uptake effect in skeletal muscle	*I. foliacea*	[135]
Polyphenolic-rich extract	α-Glucosidase inhibitor	*Ascophyllum nodosum*	[136]
Phlorotannin-rich extract	Postprandial hyperglycemia-lowering	*A. nodosum*	[137]
		*Fucus vesiculosus*	[137]
Polyphenolic-rich extract	Glucose uptake in skeletal muscle	*E. cava*	[138]
Dieckol-rich extract	Improvement of insulin sensitivity	*E. cava*	[139]
Polyphenolic-rich extract	Improvement of insulin sensitivity	*I. okamurae*	[140]

**Table 2:** Preclinical trials with marine macroalgae-derived anti-diabetic compounds. Adapted with permission from Ref. [118]. Copyright 2018 Elsevier.

Macroalgae	Major Compound	Effects	References
**Brown Algae**			
*Pelvetia siliquosa*	Fucosterol	Inhibition of blood glucose level and glycogen degradation	[122]
*Pelvetia babingtonii*	Methanol extract	α-Glucosidase inhibition and suppression of postprandial hyperglycemia	[123]
*Ecklonia stolonifera*	Polyphenols	α-Glucosidase inhibition; suppression of the increase in plasma glucose	[44]
	Phlorotannins	PTP1B and α-glucosidase inhibition	[131]
	Fucosterol	RLAR, HRAR, PTP1B, α-glucosidase activities, and AGE formation inhibition	[141]
*Eisenia bicyclis Ecklonia stolonifera*	Dieckol Eckol 7-Phloroeckol Phlorofucofuroeckol-A	α-Glucosidase, and PTP1B	[131]
*Ecklonia cava*	Dieckol 7-Phloroeckol Phlorofucofuroeckol-A 6,6-Bieckol Fucodiphloroethol-G	Activation of both AMPK and Akt signal pathways; improvement of insulin sensitivity; α-Glucosidase and α-amylase inhibition	[130]
*Ecklonia kurome*	Phlorotannins	α-Amylase inhibition; amelioration of hyperinsulinemia	[142]
*Laminaria japonica*	Polysaccharides	Reduced fasting blood glucose; increased the levels of insulin and amylin	[55]
	Butyl-isobutyl-phthalate	α-Glucosidase inhibition	[143]
*Sargassum ringgoldianum*	Polyphenols	α-Amylase and α-glucosidase inhibition	[144]
*Sargassum yezoense*	Sargaquinoic acid Sargahydroquinoic acid	Enhances the transcriptional activities of PPARα and PPARγ	[145]
		Amelioration of insulin resistance	[146]
*Sargassum wightii*	Fucoidan	α-d-glucosidase inhibition	[147]
*Sargassum polycystum*	Extract	Increasing insulin sensitivity	[117]
*Sargassum hemiphyllum*	Fucoxanthin	α-Amylase and α-glucosidase inhibition, and insulin release enhancement	[148]
*Sargassum thunbergii*	Thunberol	PTP1B inhibition	[149]
*Sargassum coreanum*	Extract	Alteration of the hepatic glucose metabolic enzyme activities and improvement of insulin resistance	[150]
*Undaria pinnatifida*	Fucoxanthin	HRAR, RLAR, PTP1B inhibition, and AGE formation	[151]
		Improve insulin signaling	
*Eisenia bicyclis*	Phlorotannins	Inhibition of AGEs and α-amylase	[152]
	Fucoxanthin	Inhibition of RLAR, HRAR, PTP1B activities and AGE formation	[151]
	Fucosterol	Inhibition of RLAR, HRAR, PTP1B, α-glucosidase activities, and AGE formation	[141]
*Ascophyllum nodosum*	PhlorotanninsFucoidan	α-Amylase and α-glucosidase inhibition	[153]
*Ishige okamurae*	Diphlorethohydroxycarmalol	α-Amylase and α-glucosidase inhibition	[133]
	Extract	Alteraation of the hepatic glucose metabolic enzyme activities, and improvement of insulin resistance	[140]
*Ishige foliacea*	Octaphlorethol A	Increase in GLUT4-mediated glucose utilization via activation of AMPK in muscle	[154]
**Red Algae**			
*Kappaphycus alvarezii, Eucheuma denticulatum*	Extract	Inhibitory activity towards α-amylase	[155]
*Gracilaria lemaneiformis*	Polysaccharide	Inhibitory activity towards α-glucosidase	[156]
*Gelidim amansii*	Ethanol extract	Significant decrease of plasma glucose	[157,158]
*Porphyra yezoensis*	Porphyran	Increase of adiponectin levels	[159]
**Green Algae**			
*Ulva rigida*	Ethanol extract	Regeneration of β-cells and/or potentiate the insulin resistance	[127]
*Ulva fasciata*	Sulfated polysaccharides	Reduce blood glucose level, and restore hepatic glycogen content	[160]
*Ulva lactula*	Polysaccharides	α-amylase, maltase, and sucrase inhibition; Delay glucose absorption	[125]

**Table 3:** The bromophenols from red algae as enzyme inhibitors linked to diabetes mellitus. Adapted with permission from Ref. [118]. Copyright 2018 Elsevier.

*Grateloupia elliptica*	2,4,6-Tribromophenol	α-Glucosidase inhibition	[146]
	2,4-Dibromophenol		
*Laurencia similis*	3′,5′,6′,6-Tetrabromo-2,4-dimethyldiphenyl ether	PTP1B inhibition	[161]
	1,2,5-Tribromo-3-bromoamino-7-bromomethylnaphthalene		
	2,5,8-Tribromo-3-bromoamino-7-bromomethylnaphthalene		
	2,5,6-Tribromo-3-bromoamino-7-bromomethylnaphthalene		
	2′,5′,6′,5,6-Pentabromo-3′,4′,3,4-tetramethoxybenzo-phenone		
	Bis-(2,3-dibromo-4,5-dihydroxybenzyl) ether		
*Odonthalia corymbifera*	Bis-(2,3-dibromo-4,5-dihydroxybenzyl) ether	α-Glucosidase inhibition	[162]
	2,3-Dibromo-4,5-dihydroxybenzyl alcohol		
	2,3-Dibromo-4,5-dimethoxybenzyl methyl ether		
	4-Bromo-2,3-dihydroxy-6-hydroxymethylphenyl 2,5-dibromo-6-hydroxy-3-hydroxymethylphenyl ether		
	4-Bromo-2,3-dimethoxy-6-methoxymethylphenyl 2,5-dibromo-6-methoxy-3-methoxymethylphenyl ether		
	4-Bromo-2,3-dimethoxy-6-methoxymethylphenyl 2,5-dibromo-6-methoxy-3-methoxymethylphenyl ether		
	3-Bromo-4,5-dimethoxybenzyl methyl ether		
*Polyopes lancifolia*	Bis-(2,3-dibromo-4,5-dihydroxybenzyl) ether	α-Glucosidase inhibition	[163]
Polysiphonia morrowii	3-Bromo-4,5-dihydroxybenzyl alcohol	α-Glucosidase inhibition	[164]
	3-Bromo-4,5-dihydroxybenzyl methyl ether		
*Rhodomela confervoides*	Bis-(2,3-dibromo-4,5-dihydroxybenzyl) methane	Potent PTP1B inhibition	[165]
	3-Bromo-4,5-bis(2,3-dibromo-4,5-dihydroxybenzyl)-1,2-benzene-diol		[166]
	3,4-Dibromo-5-(2-bromo-3,4-dihydroxy-6-(isopropoxymethyl)benzyl)benzene-1,2-diol		
	2,2′,3,3′-Tetrabromo-4,4′,5,5′-tetra-hydroxydiphenyl methane		[167]
	2,2′,3-Tribromo-3′,4,4′,5-tetrahydroxy-6′-ethyloxy-methyldiphenyl methane		
*Symphylocladia latiuscula*	2,3-Dibromo-4,5-dihydroxybenzyl methyl ether	PTP1B inhibition	[168]
	3,5-Dibromo-4-hydroxybenzoic acid		
	2,3,6-Tribromo-4,5-dihydroxymethylbenzene		
	2,3,6-Tribromo-4,5-dihydroxybenzaldehyde		
	2,3,6-Tribromo-4,5-dihydroxybenzyl methyl ether		
	Bis-(2,3,6-tribromo-4,5-dihydroxyphenyl) methane		
	1,2-Bis-(2,3,6-tribromo-4,5-dihydroxyphenyl)-ethane		
	1-(2,3,6-Tribromo-4,5-dihydroxybenzyl)-pyrrolidin-2-one		
	2,3,6-Tribromo-4,5-dihydroxybenzyl alcohol	α-Glucosidase inhibition	[164]

**Table 4:** Nanoencapsulable algal compounds against neurodegenerative disorders.

Marine Algae Species	Compounds of Interest	Model	Pharmacological Effects	References
**Brown Algae**				
*Dictyopteris undulata*	Sesquiterpene, zonarol	In vitro	Activates the Nrf2/ARE pathway, induces phase-2 enzymes, and protects neuronal cells from oxidative stress	[211]
*Eisenia bicyclis*	Phlorotannins	In vitro	Inhibits AChE at IC_50_ = 4.8 mg.mL^−1^	[212]
			Suppression of BACE-1 enzyme activity at IC_50_ = 5.35 µM	[213]
			Decreased Aβ-induced cell death at IC_50_ = 800 µM	[214]
*Ecklonia cava*	Dieckol, phlorofucofuroeckol	In vivo	Improvement of memory, and possible involvement in AChE inhibition	[207]
	Triphlorethol-A		Anti-oxidative activity, scavenging activity against ROS and DPPH via activation of ERK protein	[207]
	Phlorotannins	In vitro	Scavenging activity against hydroxyl, superoxide, and peroxyl radicals at IC_50_ = 392.5, 115.2, and 128.9 µM, respectively	[215]
		In vivo	Potentiated pentobarbital-induced sleep at >50 mg.kg^−1^	[216]
			Neuroprotective effects against H_2_O_2_-induced oxidative stress in murine hippocampal HT22 cells at IC_50_ = 50 µM	[217]
	Phloroglucinol	In vivo	Reduces the toxicity ROS induced by hydrogen peroxide at IC_50_ = 10 µg.mL^−1^	[218]
	Eckol	In vitro, In vivo	Inhibits BChE IC_50_ = 29 µM	[219]
	7-phloroeckol)	In vitro, In vivo	Inhibits BChE at IC_50_ = 0.95 µM	[219]
*Ecklonia kurome*	Acidic oligosaccharide sugar chain (AOSC)	In vitro	Blocks the fibril formation of Aβ at IC_50_ = 100 µg.mL^−1^	[220]
*Ecklonia maxima*	Phlorotannins	In vitro	Inhibits AChE at IC_50_ = 62.61 to 150.80 µg.mL^−1^	[221]
*Ecklonia stolonifera*	Phlorotannins (dieckol, eckstolonol, eckol 2-phloroeckol, 7-phloroeckol, phlorofucofuroeckol A)	In vitro	Inhibits AChE at IC_50_ = 4.89 to 42.66 µM Inhibits BuChE at IC_50_ = 136.71 to 230.27 µM	[222]
	Sterol (fucosterol)	In vitro	Inhibits BChE at IC_50_ = 421.72 µM	[222]
*Fucus vesiculosus*	Fucoidan	In vitro	Blocks microglial uptake of fDNA at only 40 ng.mL^−1^	[200]
		In vivo	Inhibits superoxide radicals, hydroxyl radicals, and lipid peroxidation at IC_50_ = 0.058, 0.157, and 1.250 mg.mL^−1^	[223]
			Neuroprotective through iNOS	[224]
			Inhibits TNF-α and IFN-γ-stimulated NO production via p38 MAPK, AP-1, JAK/STAT, and IRF-1	[225]
			Inhibits beta-amyloid induced microglial clustering at IC_50_ = 10 µM	[226]
	Phlorotannins	In vivo	Suppresses the overproduction of intracellular ROS induced by hydrogen peroxide at IC_50_ = 0.068 mg.mL^−1^	[227]
*Marginariella boryana*	Sulfated fucans	In vitro	Prevents the accumulation of Aβ	[228]
*Ishige okamurae*	Diphlorethohydroxycarmalol (DPHC)	In vivo	Neuroprotection against hydrogen peroxide (H_2_O_2_)-induced oxidative stress in murine hippocampal neuronal cells at IC_50_ = 50 µM	[67]
	Phlorotannins	In vitro	Inhibits AChE at IC_50_ = 46.42 µM Inhibits BChE at IC_50_ = 110.83 µM	[67]
*Padina gymnospora*	Fucoxanthin	In vivo	Anti-oxidative activity, reduces lipid peroxidation in rats at IC_50_ = 0.83 µM	[229]
*Papenfussiella lutea*	Sesquiterpenes	In vivo	Inhibiting AChE at IC_50_ = 65 µM	[228]
*Saccharina japonica*	Fucoidan	In vivo	Reduces the toxicity of H_2_O_2_ in PC12 cells via activation of PI3K/Akt pathway	[230]
*S. japonica*	Fucoidan	In vivo	Inhibits microglia, inhibits LPS-induced NO production via suppression of p38 MAPK and ERK phosphorylation at IC_50_ = 125 µg.mL^−1^	[231]
*Sargassum fulvellum*	Pheophytin A	In vivo	Produce neurite outgrowth, at IC_50_ = 3.9 µg.mL^−1^ in PC12 cells	[232]
*Sargassum macrocarpum*	Carotenoids, sargaquinoic acid, and sargachromenol	In vivo	Promotes neurite outgrowth activity and survival of PC-12 cells and neurite outgrowth through activation of cAMP and MAP kinase pathways at IC_50_ = 9 µM	[223]
*Sargassum micracanthum*	Plastoquinones	In vivo	Anti-oxidative activity, lipid peroxidation at IC_50_ = 0.95–44.3 µg.mL^−1^	[233]
*Hijikia fusiformis*	Fucoxanthin	In vitro	Anti-oxidative activity, DPPH radical scavenging	[53]
*Sargassum fusiforme*	Fucoidan	In vivo	Ameliorates learning and memory deficiencies, and potential ingredient for treatment of Alzheimer’s disease	[234]
*Sargassum horneri*	Total sterols, β-sitosterol	In vivo	Antidepressant effect	[235]
*Sargassum sagamianum*	Sargaquinoic acid, sargachromenol	In vitro	Inhibits AChE IC_50_ = 23.2 and 32.7 µM, respectively, inhibits BuChE at IC_50_ = 26 µM (for sargaquinoic acid)	[236]
*Sargassum siliquastrum*	Meroditerpenoids	In vitro	Radical-scavenging activity as well as weak inhibitory activities against sortase A and isocitrate lyase	[237]
*Scytothamnus australis*	Sulfated fucans	In vivo	Prevents the accumulation of Aβ	[228]
*Splachnidium rugosum*	Sulfated fucans	In vivo	Prevents the accumulation of Aβ	[228]
*Turbinaria decurrens*	Fucoidan	In vivo	Potential neuroprotective effects in Parkinson’s disease	[238]
*Undaria pinnatifida*	Glycoprotein	In vivo	AChE, BChE, and BACE1 inhibitory activities with IC_50_ values of 63.56, 99.03, and 73.35 µg.mL^−1^, respectively	[239]
*Zonaria spiralis*	Spiralisone A, Chromone 6	In vitro	Kinases inhibitory to CDK5/p25, CK1δ, and GSK3β at IC_50_ = 10.0, <10 µM, and <10 µM, respectively	[240]
**Red Algae**				
*Chondracanthus acicularis*	Lambda-carrageenan	In vitro	Inhibits superoxide radicals, hydroxyl radicals, and lipid peroxidation at IC_50_ = 0.046, 0.357, and 2.267 mg.mL^−1^, respectively	[223]
*Chondrophycus undulatus*	Floridoside	In vivo	Suppresses pro- inflammatory responses in microglia by markedly inhibiting the production of nitric oxide (NO) and reactive oxygen species (ROS) at IC_50_ = 10 µM	[241]
*Eucheuma denticulatum*	Iota-carrageenan	In vitro	Inhibits superoxide radicals, hydroxyl radicals, and lipid peroxidation at IC_50_ = 0.332, 0.281, and 0.830 mg.mL^−1^, respectively	[223]
*Gelidiella acerosa*	Phytol	In vitro, in vivo	Antioxidant activities at IC_50_ = 25–125 µg.mL^−1^	[193]
*Kappaphycus alvarezii*	Kappa-carrageenan	In vitro	Inhibits superoxide radicals, hydroxyl radicals, and lipid peroxidation at IC_50_ = 0.112, 0.335, and 0.323 mg.mL^−1^, respectively	[223]
*Ochtodes secundiramea*	Halogenated monoterpenes	In vitro	Inhibits AChE at IC_50_ = 400 µg mL^−1^	[242]
*Porphyra/Pyropia* sp.	Phycoerythrobilin	In vitro	Antioxidant activity at IC_50_ = 0.048 mmol.g^−1^	[243]
*Rhodomela confervoides*	Bromophenols	In vitro	Antioxidant activity at IC_50_ = 5.22–23.60 µM	[166]
*Rhodomelopsis africana*	Phenolic compounds, Flavonoids	In vitro	Inhibits AChE at IC_50_ = 0.12 mg.mL^−1^	[244]
**Green Algae**				
*Caulerpa racemosa*	Bisindole alkaloid (A and B), α-tocospirone, Sterol (23E)-3βhydroxystigmasta-5,23dien28-one	In vivo	Increase 5.5% of cell viability in SH-SY5Y cells, inhibits AChE at IC_50_ = 5.5 mg.mL^−1^	[245]
*Codium capitatum*	Phenolic compounds, Flavonoids	In vitro	Inhibits AChE at IC_50_ = 0.11 mg.mL^−1^	[246]
*Codium duthieae*	Phenolic compounds, Flavonoids	In vitro	Inhibits AChE at IC_50_ = 0.14 mg.mL^−1^	[246]
*Codium fragile*	Clerosterol	In vivo, in vitro	Exhibits reducing activity to COX-2, iNOS, and TNF-α at IC_50_ = 3 µg.mL^−1^	[247]
*Halimeda cuneata*	Phenolic compounds, Flavonoids	In vitro	Inhibits AChE at IC_50_ = 0.07 mg.mL^−1^	[246]
*Ulva pertusa*	Sulfated polysaccharides	In vitro	Scavenging activity for superoxide radicals	[52]
*Ulva fasciata*	Phenolic compounds, Flavonoids	In vitro	Inhibits AChE at IC_50_ = 0.07 mg.mL^−1^	[246]
*Ulva prolifera*	Pheophorbide A	In vitro	Antioxidant activity at IC_50_ = 71.9 µM	[52]

## Data Availability

Not applicable.

## Abbreviations

AChE	Acetylcholesterinase
AD	Alzheimer’s disease
As	Arsenic
Ca	Calcio
CE	Capillary electrophesis
CNS	Central nervous system
Cu	Copper
CVD	Cardiovascular disease
DAD	Diode array detection
DHA	Docosahexanoic acid
DM	Diabetes mellitus
DPPH	2,2-diphenyl-1-picrylhydrazyl
ESI	Electrospray ionisation
EPA	Eicosapentaenoic acid
FAs	Fatty acids
FAME	FA methyl esther
FID	Flame ionization detector
FDA	Food and Drug Administration
Fe	Iron
FTIR	Fourrier-transform infrared
GC	Gas chromatography
HPLC	High-performance liquid chromatography
I	Iodine
K	Potassium
LDL	Low-density lipoprotein
LPS	Lipopolysaccharides
MAA	Mycosporine-like amino acid
MAPK	Mitogen-activated protein kinase
MDR	Multidrug resistance
Mg	Magnesium
Mn	Manganese
MMPs	Matrix metalloproteinases
MNPs	Metallic NPs
MS	Mass spectrometry
Na	Sodium
NMR	Nuclear magnetic resonance
NPs	Nanoparticles
OS	Oxidative stress
P	Phosphorus
PC	Phycocyanin
PD	Parkinson’s disease
PE	Phycoerythrin
PUFAs	Polyunsaturaed fatty acids
ROS	Reactive oxygen species
SFAs	Saturated FAs
TLC	Thin-layer chromatography
UV–Vis	Ultraviolet–visible
VEGF	Vascular endothelial growth factor
WHO	World Health Organization
Zn	Zinc
